# Are All Competencies Equal in the Eyes of Residents? A Multicenter Study of Emergency Medicine Residents’ Interest in Feedback

**DOI:** 10.5811/westjem.2016.11.32626

**Published:** 2016-12-15

**Authors:** Suzanne Bentley, Kevin Hu, Anne Messman, Tiffany Moadel, Sorabh Khandelwal, Heather Streich, Joan Noelker

**Affiliations:** * Icahn School of Medicine at Mount Sinai, Elmhurst Hospital Center, Department of Emergency Medicine, Department of Medical Education, New York, New York; † Icahn School of Medicine at Mount Sinai, Department of Emergency Medicine, New York, New York; ‡ Wayne State University School of Medicine, Department of Emergency Medicine, Detroit, Michigan; § Yale School of Medicine, Department of Emergency Medicine, New Haven, Connecticut; ¶ The Ohio State University, Department of Emergency Medicine, Columbus, Ohio; || University of Virginia, Department of Emergency Medicine, Charlottesville, Virginia; # Washington University in St. Louis, Department of Medicine, Division of Emergency Medicine, St. Louis, Missouri

## Abstract

**Introduction:**

Feedback, particularly real-time feedback, is critical to resident education. The emergency medicine (EM) milestones were developed in 2012 to enhance resident assessment, and many programs use them to provide focused resident feedback. The purpose of this study was to evaluate EM residents’ level of interest in receiving real-time feedback on each of the 23 competencies/sub-competencies.

**Methods:**

This was a multicenter cross-sectional study of EM residents. We surveyed participants on their level of interest in receiving real-time on-shift feedback on each of the 23 competencies/sub-competencies. Anonymous paper or computerized surveys were distributed to residents at three four-year training programs and three three-year training programs with a total of 223 resident respondents. Residents rated their level of interest in each milestone on a six-point Likert-type response scale. We calculated average level of interest for each of the 23 sub-competencies, for all 223 respondents and separately by postgraduate year (PGY) levels of training. One-way analyses of variance were performed to determine if there were differences in ratings by level of training.

**Results:**

The overall survey response rate across all institutions was 82%. Emergency stabilization had the highest mean rating (5.47/6), while technology had the lowest rating (3.24/6). However, we observed no differences between levels of training on any of the 23 competencies/sub-competencies.

**Conclusion:**

Residents seem to ascribe much more value in receiving feedback on domains involving high-risk, challenging procedural skills as compared to low-risk technical and communication skills. Further studies are necessary to determine whether residents’ perceived importance of competencies/sub-competencies needs to be considered when developing an assessment or feedback program based on these 23 EM competencies/sub-competencies.

## INTRODUCTION

Real-time feedback during a clinical shift in the emergency department is an important component of a resident physician’s medical education and can have a profound impact on clinical practice.^1^–^4^ Despite this, many residents feel they do not get adequate or useful feedback during clinical shifts. Specific, tailored, learner-initiated feedback is crucial but rarely performed.^1^–^4^ Valid self-assessment strategies are recognized as fundamental to continuing professional competence and developing lifelong learning and improvement practices but these skills are understudied skill for development of resident physicians.^5^,^6^

The Accreditation Council for Graduate Medical Education (ACGME) introduced the Next Accreditation System (NAS) in 2012, which includes 23 emergency medicine (EM) competency / sub-competency domains, each comprised of five levels of specific developmental milestones. This model is the main assessment framework of the NAS. Physicians are expected to progress through the milestone levels of each competency / sub-competency from novice intern to expert.^2^,^7^–^10^

Various EM studies have revealed widespread dissatisfaction with feedback despite the employment of a wide variety of feedback methods. Most studies on feedback involve attending- or program leader-initiated feedback. Few have explored the theme of learner-initiated feedback. 1–4,9–11 To date, few studies have explored EM resident interest in feedback on specific competencies/sub-competencies despite the widespread use of this structured feedback mechanism.

The objective of this research project was to evaluate EM residents’ level of interest in receiving real-time feedback on each of the 23 competencies/sub-competencies. Identifying the areas of most importance to learners may be the first step in helping mitigate issues with poor feedback and giving learners more autonomy over desired feedback.

## METHODS

This was a multicenter cross-sectional study of EM residents at six ACGME-accredited academic EM residency programs in the United States. The programs span various regions of the country with three three-year and three four-year residency programs in both urban and suburban settings (Table 1). Participants were surveyed on their level of interest in receiving real-time feedback on each of the competencies/sub-competencies. Anonymous paper or computerized surveys using SurveyMonkey (a commercially available online survey creation and distribution program: http://www.surveymonkey.com) were distributed to residents of all postgraduate year (PGY) levels at each of the six training programs with a total of 272 possible resident respondents. The project was deemed exempt by the IRB at the Icahn School of Medicine at Mount Sinai followed by review at the remaining institutions.

We surveyed all residents at the six academic EM residency programs regarding their interest levels in receiving feedback by the EM attending during a clinical shift on specific topic areas covering the 23 ACGME EM competencies/sub-competencies. Surveys were distributed at each institution during the middle of the academic calendar year via paper survey and then subsequently via email to capture residents who were not able to complete paper forms. Completion of the survey was considered consent for the study. Study participation was anonymous and voluntary. We provided residents the survey questionnaire (Appendix 1a) along with milestone descriptions (Appendix 1b).

For content validity, the survey was designed to include all 23 competencies/sub-competencies. To optimize content and internal structure evidence, we created the survey instrument using an iterative editing approach. This included extensive testing among the authors for item generation, survey functionality, matching of item content to the construct, optimal item phrasing, and overall quality control. For response process validity, the survey was piloted by six EM attending physicians and six EM senior resident physicians and subsequently revised.

Residents rated their level of interest in receiving on-shift feedback on each competency/sub-competency using a six-point Likert-type response scale (1= no interest; 2= minimal interest; 3=mild interest; 4=moderate interest; 5=very interested; 6=maximal interest). We calculated average levels of interest for each of the 23 competencies/sub-competencies for all respondents and by PGY level of training. A one-way analysis of variance (ANOVA) was conducted to determine whether differences in desire for feedback existed by level of training (PGY level). To control for Type-1 error rates from multiple comparisons, we adjusted the p-value for significance using the Bonferroni correction suggested by Bland, 1995 (p=.05/23 tests= .002).^12^

## RESULTS

The overall survey response rate was 82% (223/272). Return rates and residency characteristics are detailed in Table 1. The number of survey participants was almost equivalent for PGY levels 1–3 (60 or 27% for PGY-1s, and 62 or 27.8% for both PGY-2s and 3s). The number of PGY-4 participants was considerably lower at 34 (15.2%).

One-way ANOVA analyses (Table 2) showed no statistical differences between residents at different levels of training for any of the 23 competencies/sub-competencies after adjustment with the Bonferroni correction. When looking at the differences in average ratings from all residents combined, we noticed considerable variability across the 23 competencies/sub-competencies (see Table 2). The competencies/sub-competencies with highest average ratings were received by emergency stabilization (rating: 5.47), airway management (5.35), and medical knowledge (5.08). These ratings indicate that residents are very or maximally interested in receiving feedback on these competencies/sub-competencies. Ratings on an additional nine competencies/sub-competencies would indicate that residents are very interested in feedback. These mean ratings ranged from 4.54 and 4.90. Residents indicated that they would be moderately interested in feedback on 10 competencies/sub-competencies (rated 3.61 to 4.27). Only one competency/sub-competency received a rating that would indicate that residents had mild interest: technology/EHR (3.24).

## DISCUSSION

The EM Milestones project, developed by the ACGME and the American Board of Emergency Medicine, provides residency programs with descriptive, objective criteria by which to assess a resident’s progress throughout his or her training. While program directors and academic faculty in residency programs are familiar with the milestone sub-competencies, it is less clear if residents have similar investment in the tools being used to evaluate them. Some residents may have little to no knowledge about each of the individual competencies/sub-competencies and the criteria used to differentiate various levels of performance on the milestones scale. Residents may also not internalize feedback on competencies/sub-competencies for which they feel are not relevant to them at a given time. This study aimed to assess EM residents’ interest in receiving real-time feedback on each of the 23 different EM competencies/sub-competencies.

Of the 23 competencies/sub-competencies, residents were most interested in receiving feedback on three: emergency stabilization, airway management, and medical knowledge. Compared to the other milestones, these seem to reflect the core values of the practice of EM – complicated skill sets that are high reward, if done well, and have significant impact on patient outcomes. Of these, emergency stabilization and medical knowledge encompass broad content areas covered during residency education.

There was one outlier competency on which residents were least interested in receiving feedback: technology and electronic health records. This competency had the lowest average interest rating at 3.24 out of 6, reflecting mild interest in receiving feedback. Possible explanations for why this milestone was least interesting to residents include lack of understanding of its importance in their future career, lack of perceived relevance to direct patient outcome, difficulty in receiving feedback on this work, or even perceived adequacy of prior or current feedback on this competency.

All other competencies/sub-competencies received ratings between 3.6–4.9, reflecting significant resident interest in receiving feedback on these topics. By rating all of the competencies/sub-competencies as at least mildly interesting regarding feedback, residents are validating the idea that the competencies/sub-competencies accurately represent relevant learning objectives throughout residency that are perceived as applicable to their future practice. There were no statistically significant differences between residents based on PGY level regarding their interest in milestone-based feedback, suggesting that feedback on any of the competencies/sub-competencies would be appreciated at any learner level.

Prior work suggests that a trainee’s prior experiences, confidence level, fear of appearing incompetent, and biases in cognitive reasoning processes can affect their responsiveness to feedback.^13^ Those who are learning goal-oriented may aim to prioritize feedback on topics that they feel weaker in, as they are more likely to use unsatisfactory performance as an impetus for improvement. On the contrary, learners with performance-based goals may seek to validate their own competency over their peers by seeking out favorable judgments and avoiding negative comments about one’s competence.^14^,^15^ Understanding the subtle differences in a resident’s interest in receiving feedback on each competency and the motivation behind these differences will be useful for programs going forward in their quest to provide desired, well-rounded, relevant, actionable feedback to further the development of their residents.

## LIMITATIONS

A limitation of this study is the variability in response rates across the participating institutions. The lowest survey response rate at a site was 69% while the site with the highest response rate was 93%. However, such a diverse subject population is important for allowing generalizability of aggregate resident survey responses across the larger group of EM trainees across the country.

To obtain the highest possible response rate, some residents were given a paper survey while others participated in the online survey. The different vehicles by which certain residents responded may have affected the responses given.

## CONCLUSION

Providing effective feedback to residents is essential to their education and professional growth. Residents frequently report discontent with the feedback they receive, and thus a better understanding of feedback and residents’ preferences regarding feedback may allow attending physicians to provide more useful feedback. We observed no differences between resident levels of training, suggesting that preference for feedback is unrelated to PGY level. Future areas of research in this domain include elucidating whether feedback is more effective if it involves a sub-competency of particular interest to the resident, and if sub-competencies deemed “less interesting” require particular attention to reinforce their importance in a resident physician’s career.

## Supplementary Information





## Figures and Tables

**Figure:**
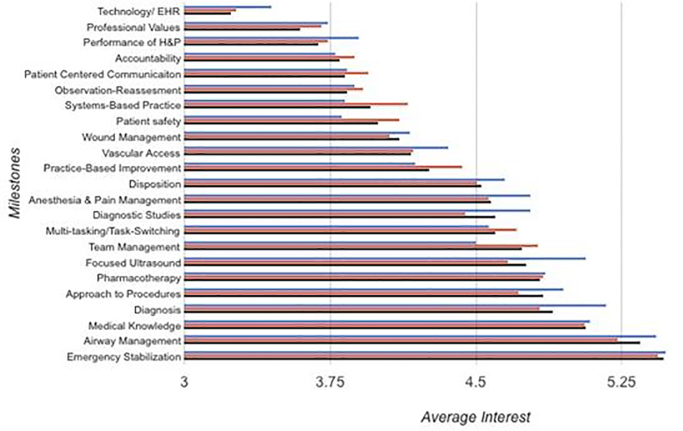
Resident feedback interest by competencies/sub-competencies.

**Table 1:** Demographic information on six emergency medicine residency programs and survey return rates for 272 emergency medicine residents from those programs.

Program	Residents/year	Total resident number	Number & percent survey return	Geographic region	Program setting	Program length	Annual patient volume
1	15	60	49 (81.7)	Northeast	Urban	4	100,000
2	12	48	35 (72.9)	Midwest	Urban	4	95,000
3	13–15	56	51 (91.1)	Northeast	Urban	4	100,000
4	12	36	25 (69.4)	Midwest	Urban	3	105,000
5	10	30	28 (93.3)	Midatlantic	Suburban	3	61,000
6	16	42	35 (83.3)	Midwest	Urban	3	80,000

**Table 2:** Descriptive statistics and results of one-way analysis of variance comparing 217 emergency medicine residents on their ratings of interest in feedback on 23 competencies/sub-competencies.

	Mean ratings (std. dev. in parentheses)				ANOVA results		
		
Competencies/sub-competencies	All (N=217)	PGY1 (N=60)	PGY2 (N=62)	PGY 3&4 (N=95)	F	df	p
Emergency stabilization	5.47 (.82)	5.48 (.77)	5.48 (.84)	5.44 (.85)	0.10	2, 214	0.90
Airway management	5.35 (0.87)	5.43 (0.87)	5.48 (0.74)	5.23 (0.94)	1.63	2, 214	0.20
Medical knowledge	5.07 (1.05)	5.09 (1.13)	5.08 (0.87)	5.06 (1.11)	0.02	2, 214	0.98
Diagnosis	4.90 (1.03)	5.17 (0.91)	4.75 (0.99)	4.83 (1.10)	2.88	2, 214	0.06
Approach to procedures	4.85 (1.13)	4.95 (1.15)	4.93 (0.92)	4.72 (1.24)	1.00	2, 214	0.37
Pharmacotherapy	4.83 (1.03)	4.86 (1.22)	4.80 (1.01)	4.85 (0.93)	0.08	2, 210	0.93
Goal-directed focused ultrasound	4.76 (1.17)	5.03 (1.13)	4.65 (1.18)	4.67 (1.16)	2.24	2, 214	0.11
Team management	4.74 (1.21)	4.50 (1.27)	4.80 (1.10)	4.82 (1.23)	1.38	2, 214	0.25
Diagnostic studies	4.60 (1.05)	4.78 (1.02)	4.54 (1.07)	4.45 (1.07)	0.92	2, 215	0.40
Multi-tasking/task-switching	4.60 (1.26)	4.57 (1.13)	4.43 (1.34)	4.71 (1.29)	0.80	2, 215	0.45
Anesthesia & pain management	4.58 (1.16)	4.78 (1.12)	4.44 (1.18)	4.57 (1.16)	1.41	2, 214	0.25
Disposition	4.53 (1.19)	4.65 (1.11)	4.46 (1.22)	4.51 (1.23)	0.46	2, 213	0.64
Practice-based improvement	4.26 (1.36)	4.19 (1.32)	4.08 (1.48)	4.43 (1.30)	1.22	2, 214	0.30
Vascular access	4.17 (1.29)	4.36 (1.29)	3.98 (1.32)	4.18 (1.26)	1.72	2, 214	0.18
Wound management	4.11 (1.28)	4.16 (1.43)	4.11 (1.19)	4.06 (1.25)	0.12	2, 214	0.89
Patient safety	4.00 (1.31)	3.81 (1.33)	4.02 (1.25)	4.11 (1.33)	0.92	2, 212	0.40
Systems-based practice	3.96 (1.27)	3.83 (1.26)	3.77 (1.31)	4.15 (1.24)	1.94	2, 214	0.15
Observation-reassessment	3.84 (1.26)	3.88 (1.39)	3.66 (1.21)	3.92 (1.20)	0.75	2, 213	0.47
Patient-centered communication	3.83 (1.35)	3.84 (1.40)	3.67 (1.35)	3.95 (1.32)	0.66	2, 214	0.52
Accountability	3.80 (1.47)	3.78 (1.63)	3.62 (1.39)	3.88 (1.41)	0.43	2, 213	0.65
Performance of H&P	3.69 (1.41)	3.90 (1.27)	3.41 (1.53)	3.74 (1.41)	1.67	2, 214	0.19
Professional values	3.60 (1.46)	3.74 (1.53)	3.34 (1.50)	3.71 (1.38)	1.20	2, 214	0.30
Technology/EHR	3.24 (1.44)	3.45 (1.38)	2.95 (1.45)	3.27 (1.46)	1.59	2, 214	0.21

* Bonferroni adjustment is used to control for Type 1 error rates. The adjusted p value for considering a mean difference statistically significant is equal to 0.05/23 = 0.002.

*ANOVA,* one-way analysis of variance; *PGY,* post-graduate year; *H&P,* history and physical; *EHR,* electronic health records
