# Supplementary material for: Are All Competencies Equal in the Eyes of Residents? A Multicenter Study of Emergency Medicine Residents’ Interest in Feedback
Source: West J Emerg Med. 2016 Dec 15;18(1):76–81. doi: 10.5811/westjem.2016.11.32626 (PMC5226767; doi:10.5811/westjem.2016.11.32626)
Supplement: Supplementary file 2 [file wjem-18-76-s002.pdf]

Milestone Descriptions

1. **Emergency Stabilization:** Prioritizes critical initial stabilization action and mobilizes hospital support services in the resuscitation of a critically ill or injured patient and reassesses after stabilizing intervention.

2. **Performance of Focused History and Physical Exam:** Abstracts current findings in a patient with multiple chronic medical problems and, when appropriate, compares with a prior medical record and identifies significant differences between the current presentation and past presentations.

3. **Diagnostic Studies:** Applies the results of diagnostic testing based on the probability of disease and the likelihood of test results altering management.

4. **Diagnosis:** Based on all of the available data, narrows and prioritizes the list of weighted differential diagnoses to determine appropriate management.

5. **Pharmacotherapy:** Selects and prescribes, appropriate pharmaceutical agents based upon relevant considerations such as mechanism of action, intended effect, financial considerations, possible adverse effects, patient preferences, allergies, potential drug-food and drug-drug interactions, institutional policies, and clinical guidelines; and effectively combines agents and monitors and intervenes in the advent of adverse effects in the ED.

6. **Observation and Reassessment:** Re-evaluates patients undergoing ED observation (and monitoring) and using appropriate data and resources, determines the differential diagnosis and, treatment plan, and disposition.

7. **Disposition:** Establishes and implements a comprehensive disposition plan that uses appropriate consultation resources; patient education regarding diagnosis; treatment plan; medications; and time and location specific disposition instructions.

8. **Multi-tasking (Task-switching):** Employs task switching in an efficient and timely manner in order to manage the ED.

9. **General Approach to Procedures:** Performs the indicated procedure on all appropriate patients (including those who are uncooperative, at the extremes of age, hemodynamically unstable and those who have multiple co-morbidities, poorly defined anatomy, high risk for pain or procedural complications, sedation requirement), takes steps to avoid potential complications, and recognizes the outcome and/or complications resulting from the procedure.

10. **Airway Management:** Performs airway management on all appropriate patients (including those who are uncooperative, at the extremes of age, hemodynamically unstable and those who

have multiple co-morbidities, poorly defined anatomy, high risk for pain or procedural complications, sedation requirement), takes steps to avoid potential complications, and recognize the outcome and/or complications resulting from the procedure.

11. **Anesthesia and Acute Pain Management:** Provides safe acute pain management, anesthesia, and procedural sedation to patients of all ages regardless of the clinical situation.

12. **Goal-directed Focused Ultrasound (Diagnostic/Procedural):** Uses goal-directed focused Ultrasound for the bedside diagnostic evaluation of emergency medical conditions and diagnoses, resuscitation of the acutely ill or injured patient, and procedural guidance.

13. **Wound Management**: Assesses and appropriately manages wounds in patients of all ages regardless of the clinical situation.

14. **Vascular Access:** Successfully obtains vascular access in patients of all ages regardless of the clinical situation.

15. **Medical Knowledge:** Demonstrates appropriate medical knowledge in the care of emergency medicine patients.

16. **Patient Safety:** Participates in performance improvement to optimize patient safety.

17. **Systems-based Management:** Participates in strategies to improve healthcare delivery and flow. Demonstrates an awareness of and responsiveness to the larger context and system of health care.

18. **Technology:** Uses technology to accomplish and document safe healthcare delivery.

19. **Practice-based Performance Improvement:** Participates in performance improvement to optimize ED function, self-learning, and patient care.

20. **Professional values:** Demonstrates compassion, integrity, and respect for others as well as adherence to the ethical principles relevant to the practice of medicine.

21. **Accountability:** Demonstrates accountability to patients, society, profession and self.

22. **Patient Centered Communication:** Demonstrates interpersonal and communication skills that result in the effective exchange of information and collaboration with patients and their families.

23. **Team Management:** Leads patient-centered care teams, ensuring effective communication and mutual respect among members of the team.
